# A Novel Wide-Range Freshwater Cyanophage MinS1 Infecting the Harmful Cyanobacterium *Microcystis aeruginosa*

**DOI:** 10.3390/v14020433

**Published:** 2022-02-20

**Authors:** Shanshan Zhang, Xiaoqi He, Lei Cao, Yigang Tong, Baohua Zhao, Wenlin An

**Affiliations:** 1 College of Life Science, Hebei Normal University, Shijiazhuang 050024, China; zhangshanshanhbtu@163.com; 2 College of Life Science and Technology, Beijing University of Chemical Technology, Beijing 100029, China; 2020201121@buct.edu.cn (X.H.); 2021201103@buct.edu.cn (L.C.); 3 Department of Scientific Research Management, National Vaccine and Serum Institute, Beijing 100176, China

**Keywords:** cyanophage, *Microcystis*, genome analysis, biological characteristics

## Abstract

*Microcystis aeruginosa*, as one of the major players in algal bloom, produces microcystins, which are strongly hepatotoxic, endangering human health and damaging the ecological environment. Biological control of the overgrowth of *Microcystis* with cyanophage has been proposed to be a promising solution for algal bloom. In this study, a novel strain of *Microcystis* cyanophage, MinS1, was isolated. MinS1 contains an icosahedral head approximately 54 nm in diameter and a 260 nm-long non-contractile tail. The phage genome consists of a linear, double-stranded 49,966 bp DNA molecule, which shares very low homology with known phages in the NCBI database (only 1% of the genome showed weak homology with known phages when analyzed by megablast). The phage contains 75 ORFs, of which 23 ORFs were predicted to code for proteins of known function, 39 ORFs were predicted to code for proteins of unknown function, and 13 ORFs showed no similarity to any protein sequences. Transmission electron microscopy and phylogenetic analysis showed that MinS1 belongs to the family *Siphoviridae*. Various experiments confirmed that the phage could infect several different orders of cyanobacteria, including Chroococcales, Nostocales, Oscillatoriales, Hormogonales, and Synechococcales, indicating that it has a very broad host range. In addition, MinS1 has no known antibiotic tolerance genes, virulence genes, and tRNAs, and it is tolerant to temperature, pH, UV, and salinity, suggesting that MinS1 has good potential for application as a biological control agent against cyanobacterial blooms. This study expands the diversity and knowledge of cyanophages, and it provides useful information for the development of novel prevention and control measures against cyanobacterial blooms.

## 1. Introduction

Water eutrophication is a global water pollution problem, which causes massive production of cyanobacteria [1,2]. The latter challenge can also lead to cyanobacteria blooms [3], which have become a common occurrence in water bodies worldwide. *Microcystis* is one of the most pervasive bloom-forming cyanobacteria in freshwater ecosystems [4,5,6], consuming large amounts of dissolved oxygen and causing water quality degradation. Its metabolites, algal toxins, are hepatotoxic, neurotoxic, reproductive toxic, genotoxic, and tumor promoting, causing great economic losses to aquaculture and posing risks to the safety of aquatic products for consumption [7,8].

Viruses infecting cyanobacteria are referred to as cyanophages, and they can play major roles in the dynamics, genetic diversity, and structure of cyanobacterial communities [9,10]. These cyanophages inhibit the production and release of algal toxins and are considered to have significant potential as biological control agents for harmful cyanobacterial blooms. Compared with the existing physical and chemical cyanobacteria control methods, which are prone to take the good with the bad and secondary pollution, they have the advantages of low cost, not harming eukaryotes and other beneficial microorganisms, and not causing secondary pollution. However, most reported cyanophages have a long lysis cycle, a narrow cyanobacteria-killing spectrum, and strong host specificity [11,12]. Compared to marine cyanophages, which have been widely studied [13], there are very limited studies available pertaining to freshwater cyanophages [14]. Current research aims to build the foundations of cyanophage-based water bloom treatment, much of which are learnt from the role that these viruses play in bloom decay, particularly concentrating on an environmentally friendly treatment therapy to combat water blooms [15,16].

In the present work, we report on the morphological and biological characteristics, as well as the genomic information, of MinS1, which was isolated from freshwater in Fujian, China. Not only is MinS1 a novel M. *aeruginosa* cyanophage, but it also has a broad range of hosts. This work provides basic data for further understanding a wide range of cyanophage hosts and, additionally, enriches the database of freshwater cyanophage genome information, which is of great research significance. This work also provides basic data underpinning freshwater cyanophage genomes, and it supports the growing interest in using freshwater cyanophages to control bloom-forming cyanobacterium.

## 2. Materials and Methods

### 2.1. Cyanophage Isolation and Purification

The 30 mL samples of surface cyanobacterial bloom water used for cyanophage separation were collected from the Mayang Stream (24°32′37.81″ N 117°46′26.40″ E), in Fujian, China, and centrifuged at 12,000 g for 20 min at 4 °C. The temperature, date, and time of the water collection were 20 °C, 23 November 2020, at 10:17 a.m., respectively. The supernatant was filtered through nitrocellulose membranes with pore sizes of 0.45 μm and 0.22 μm. The filtrate was then added to a ten-times-larger volume of algal solution of the logarithmic growth M. *aeruginosa* strain FACHB-905, which can produce microcystin. The host concentration used in the experiment was 3.4 × 10^6^ CFU/mL. Then, the solution was mixed and incubated in a light incubator (Ningbo Jiangnan instrument Factory, model number: GXZ-280B) at 25 °C, 2000 Lux, with a 12 h:12 h light–dark cycle for about three days until yellowing was evident. The procedure was repeated three times. The resulting lysate was then serially diluted ten-fold using BG-11 medium (Qingdao Haibo Biotechnology Co., Ltd. product number: HB8793) and used in double-layer agar plate spread experiments [17]. Once the growth of phagocytic spots was observed, several individual spots were dug up and suspended in 5 mL of FACHB-905 solution during the logarithmic phase. The aforementioned phagocytic spot experiment was repeated after yellowing until phagocytic spots consisting of uniform shapes and sizes had formed on each plate.

### 2.2. Transmission Electron Microscopy

A total of 30 mL of phage lysate was centrifuged at 6000× *g* for 10 min, and the supernatant was filtered through a 0.22 μm microporous filter. Thereafter, the supernatant was added to a 50 mL ultracentrifuge tube, and a 30% (*w:v*) sucrose solution was slowly injected from the bottom of the sample using a pipette and then centrifuged at 4 °C at 35,000× *g* for 1 h. The phage precipitate was resuspended into 200 μL of 1 × TNE (10 mmol/L Tris-HCl, 1 mmol/L EDTA, 100 mmol/L NaCl) in order to obtain the ultra-purified phage. Next, 1 μL of the ultra-purified phage (remaining samples could be stored directly at 4 °C) was dropped onto a grid surface, 2% uranyl acetate negative staining was undertaken, and then the excess staining solution was immediately removed. Phage morphology was then examined under a 100 kV transmission electron microscope (Hitachi H-7650, Tokyo, Japan) [18].

### 2.3. Host Range

Thirty cyanobacterial strains (containing five orders and eight families) were cultured in our laboratory, identified using 16S rRNA genes, and cultured to a concentration of approximately 3.4 × 10^6^ PFU/mL in logarithmic stage to serve as test hosts. All information about test hosts can be found on Freshwater Algae Culture Collection at the Institute of Hydrobiology, and the culture conditions were the same as above. Then, 250 μL of algal solution and 50 μL of phage lysate (experimental group) were added into 96-well plate wells (Thermo Scientific, Waltham, MA, USA), incubated in a light incubator at 25 °C, 2000 Lux, with a 12 h:12 h light–dark cycle. An equal volume of 250 µL of algal solution and 50 µL of BG11 medium was also added into the other wells (control group); these were incubated under the same conditions as indicated above. Three parallel experiments were set up for each group. After 7 days, the lytic nature of the phage was determined by measuring the OD680, as well as the number, density, integrity, transparency, and edge clarity of the cells when observed using light microscopy.

In addition, 30 cyanobacterial strains were incubated until their log phase was achieved, and then they were spread in double-layer plates. Then, 5 μL of each phage solution was spotted onto each plate, which was dried and placed upside down in a light incubator in order to observe the appearance of any spots [19].

### 2.4. One-Step Growth Curve

One-step growth curve experiments were performed according to existing methods, with some minor modifications. A total of 5 mL of purified phage supernatant was added to 25 mL of the host strain and stirred for 30 min at 25 °C, 100 rpm on a shaker, followed by centrifugation at 6000 *g* for 10 min. The supernatant was discarded, and the precipitate was washed twice using PBS and then suspended in 30 mL of BG11 medium; this was then incubated in a lighted incubator. Samples were collected at 0, 6, 12, 18, 24, 30, 36, 42, 48, 54, 60, 66, and 72 h. All of the 1 mL samples were centrifuged (6000 *g*, 10 min) and filtered (using 0.45 μm and 0.22 μm filters), and titration was undertaken using the double-layer agar plate method. The burst size was calculated as the ratio of the number of free phages at the end of the logarithmic period to the number of infected host cyanobacteria cells at the beginning of the latent period. The experiment was repeated in triplicate.

### 2.5. Phage Stability under Environmental Stress

Cyanobacterial cleavage by cyanophages within natural environments is affected by water temperature, pH, salinity, ions, and UV. To examine the tolerance levels of the phage to these conditions, a published method was used with appropriate modifications [20,21]. The stability of the phage under different temperature treatments was analyzed by taking 1 mL of purified phage solution at different temperatures (0, 25, 40, 60, and 80 °C) at both 30 min and 60 min. Phage stability at different pH levels (2, 3, 4, 5, 6, 7, 8, 9, 10, 11, 12) was evaluated by adding the purified phage solution to different pH buffers for 1 h at 25 °C. To evaluate the effects of differing salt conditions on the phage, purified phage pellets were incubated at different NaCl concentrations (0%, 5%, 10%, 15% and 20%) at 25 °C for 1 h. Phage metal ion sensitivity was determined by incubating the phage in different metal ions (Ca^2+^, Mg^2+^, Fe^2+^), at a final concentration of 10 mM, for 25 min at 25 °C. Phage sensitivity to UV was assessed by irradiating purified phage particles under a UV lamp (253.7 nm) for different amounts of time (0, 20, 40, 60, and 80 min).

Untreated phage solution was used as a control, and each of the treatments was repeated three times. The solutions were all titrated using the double-layer agar plate method. In order to corroborate the double-layer agar plate results, the treated phage from each group was added to the FACHB-905 host cyanobacteria to measure the OD680 values of the mixture after a 4-day incubation period. In these experiments, the original phage titer was 6.3 × 10^6^ PFU/mL, and the host concentration was 3.9 × 10^7^ PFU/mL. One-way ANOVA and SPSS 13.0 Duncan’s new multiple range test were used to determine statistical significance. GraphPad Prism (8.0.2) was used for curve plotting and data statistical analysis.

### 2.6. DNA Extraction, Sequencing and Assembly

Phage DNA was extracted using a phenol–chloroform extraction method [22]. A 2 × 300 bp paired-end DNA library was constructed according to the manufacturer’s instructions for the NEBNext Ultra™ II DNA Library PrepKit for Illumina, and the phage was genomically sequenced using the Illumina MiSeq (San Diego, CA, USA) sequencing platform. Low-quality (Q value < 20) reads and adaptors were filtered out using fastp. Clean reads were assembled using SPAdes 3.13.0 software (http://cab.spbu.ru/software/spades/, 29 July 2021) [23]. Analysis of the phage ends was performed using an established method [24].

### 2.7. Genome Annotation and Phylogenetic Analysis

Gene prediction was initially executed using the Rapid Annotation using Subsystem Technology (RAST) annotation server (http://rast.nmpdr.org/, 13 August 2021) and then identified by searching through BLASTp (https://blast.ncbi.nlm.nih.gov/Blast.cgi, 3 January 2022), HMMER (https://www.ebi.ac.uk/Tools/hmmer/search/hmmscan, 3 January 2022), and HHpred (https://toolkit.tuebingen.mpg.de/tools/hhpred, 3 January 2022) web servers [25]. Genes were compared with other sequences at the nucleotide and amino acid levels using the NCBI BLAST tool (https:/blast.ncbi.nlm.nih.gov/, 6 January 2022). A phylogenetic tree based on whole-genome sequences was constructed using the ViPTree (https://www.genome.jp/viptree/, 6 January 2022) [26] and VIRIDIC tools (http://viridic.icbm.de/, 6 January 2022).

## 3. Results and Discussion

### 3.1. Isolation and Morphology of Cyanophage MinS1

**Figure 1:**
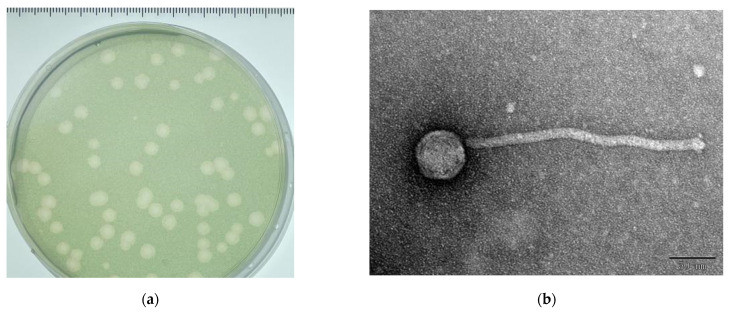
Morphology of the plaques and MinS1. MinS1 (**a**) plaques and (**b**) a transmission electron micrograph. Scale bar represents 50 nm.

M. *aeruginosa* cyanophage MinS1 (MinS1) [27] was isolated from the surface freshwater samples obtained from the Mayang Stream, located in Fujian, China, which had an outbreak of cyanobacterial blooms (more specific information is shown in Table S1). The plaques resulting from MinS1 lysis appeared clear and circular, with diameters of 3–4 mm, following a 4-day incubation (25 °C, 2000 Lux, 12 h:12 h light–dark cycle) on host algal plates at a multiplicity of infection (MOI) of 0.1 (Figure 1a). TEM of the purified phage particles showed that MinS1 had an isometric hexagon head measuring about 54 nm in diameter and a non-retractable long tail around 260 nm long (Figure 1b). The head diameter of all *Microcystis* cyanophages ranged from 42 nm to 100 nm, with MinS1 having the second smallest head among these phagosomes. MinS1 is morphologically most similar to Mic1 [28] and has a non-contractible tail second only to Mic1 (400 nm) in length. Based on its morphology, and comparisons to the current International Committee on Taxonomy of Viruses (ICTV) classification system, MinS1 therefore belongs to the family *Siphoviridae* from the order Caudovirales. MinS1 is conserved at the China General Microbiological Culture Collection Center (CGMCC) under CGMCC No. 23089.

### 3.2. Life Cycle

**Figure 2:**
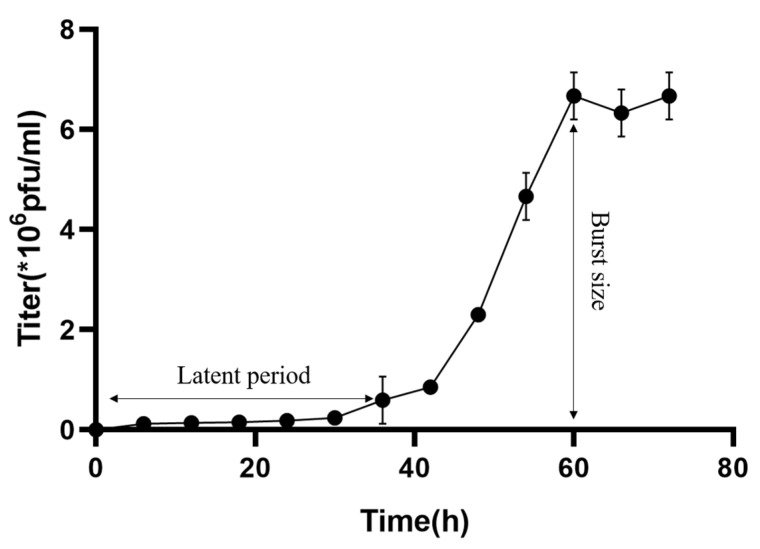
One-step growth curve for the MinS1 cyanophage. Data represent the mean and standard deviation of the independent triplicate experiments.

To understand the growth kinetics of MinS1 in the host M. *aeruginosa* strain FACHB-905, one-step growth curves of MinS1 were performed using a modified soft-agar overlay method [29]. The results show that the MinS1 latent period lasted 36–42 h and was followed by a plateau period after 60 h (Figure 2). Burst size is often considered for phage usage as biocontrol because it indicates the lytic ability of the phage. The MinS1 burst size was around 34 PFU per cell, and the total time duration of the one-step growth curve experiment lasted 72 h. The latent period and burst size of *Microcystis* cyanophages appeared to be highly variable, ranging from 6 h to 108 h and from 28 to 127 PFU per cell. It is important to note that different methods were used to count viral abundance. To date, only five strains of the Microcystis phage with one-step growth curves have been reported in the literature (Table 1), three of which were hosted by the same host as MinS1, M. aeruginosa. The latent period for MaMV-DC was 24–48 h, with an outbreak of 80 PFU per cell [30]. Ma-LBP had a latent period of 11.2 h, followed by a burst size of 80 PFU per cell [31]. Another *Microcystis* cyanophage, Ma-LMM01, revealed a latent period of 6–12 h with a burst size of 50–120 PFU per cell [32]. In general, burst size is considered to be influenced by many factors, including bacterial/viral size, metabolic activity of the host, and phage and host characteristics [33]. A correlation between outbreak size and the environmental trophic state has also been proposed [34,35]; however, this concept still requires further validation. Similar to the physiological and biological characteristics of phages, the features that influence the burst size of each phage should not be neglected.

### 3.3. Host Range

Host infectivity tests showed that the phage MinS1 had polyvalent infectivity, as shown by the infection of 19 out of 30 cyanobacteria strains tested, containing five orders, *Chroococcales, Nostocales, Oscillatoriales, Hormogonales*, and *Synechococcales* (Table 2 and Supplementary Figure S1). This represents the broadest host range of the six reported *Microcystis* cyanophage strains (Ma-LMM01, MaMV-DC, Ma-LBP, vB_MelS-Me-ZS1, phiMa05 [36], and Mic1). This wide host range indicates that MinS1 has potentially significant applications, as water blooms are usually caused by multiple cyanobacteria [37]. Generally, *Myoviruses* have shown the broadest host range among the three families comprising the tailed viruses, whereas *Podoviruses* have the narrowest range [38]. Interestingly, a narrow host range was observed for *Myoviruses* Ma-LMM01, MaMV-DC, and phiMa05 and a *Siphoviruses* Mic1, whereas *Siphoviruses* vB_MelS-Me-ZS1 had a wide host range infecting 12 of the 15 host algal strains. Moreover, the host range of Ma-LBP has not previously been reported; it is a member of the *Podoviridae* family (Table 1). Host range may be influenced by the number of tested host algal strains. Moreover, the cyanophages’ interaction in the environment, for example, their dynamics during cyanoHABs, may also affect their ability to infect algal strains. However, undeniably, this confirms the specificity and complexity of cyanophage–host interactions and the diversity within the *Microcystis* phages.

**Table 1:** A full list of Microcystis cyanophages, including cyanophage MinS1.

Phage Name	Latent Period (h)	Burst Size (PFU/Cell)	Classification	Length (bp)	Accession	Host Range	Reference
1 MaMV-DC	24–48	80	Myoviridae	169,223	KF356199.1	Strain specific	[31]
2 Ma-LMM01	6–12	50–120	Myoviridae	162,109	AB231700.1	Strain specific	[33]
3 Ma-LBP	11.2	28	Podoviridae	-	-	-	[32]
4 vB-MelS-Me-ZS1	108	-	Siphoviridae	49,665	MK069556	12/15	[5]
5 phiMa05	24	127	Myoviridae	273,876	MW495066.1	Strain specific	[37]
6 Mic1	-	-	Siphoviridae	92,627	MN013189.1	Strain specific	[29]
7 MinS1	36–42	34	Siphoviridae	49,966	MZ923504	19/30	-

Symbols: “-” means the information was not reported.

The interactions between cyanobacterial strains and environmental microorganisms and the co-evolution of phagosomes and cyanobacteria make it impossible for phagosomes to kill all of them, even if they are broad-spectrum phagosomes, and some of them will be selectively retained. In addition, the interaction between a phage with a variable host range and cyanobacteria can catalyze mutations and tolerant strains, promoting continuous turnover and, thus, reaching a dynamic equilibrium. Therefore, the application of broad-spectrum phages does not destroy biodiversity. No reduction in species diversity has occurred during natural evolution due to the presence of phages.

### 3.4. Thermolability, pH, UV, Ions, Sensitivity, and Saline Stability

**Figure 3:**
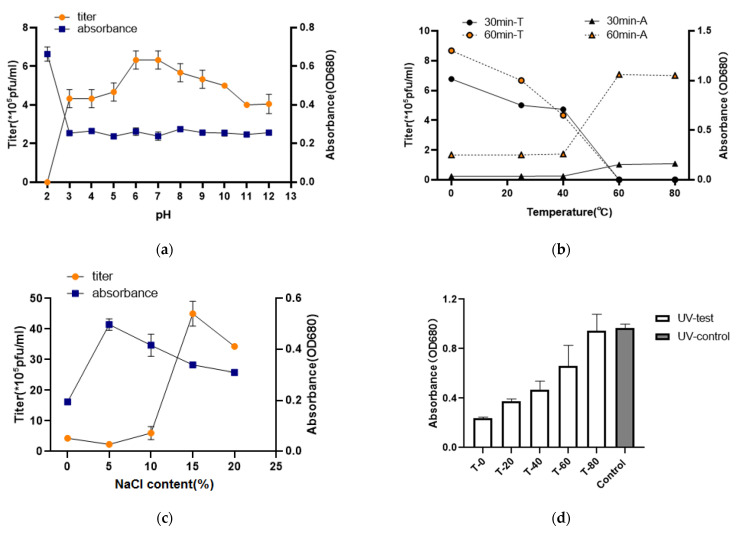

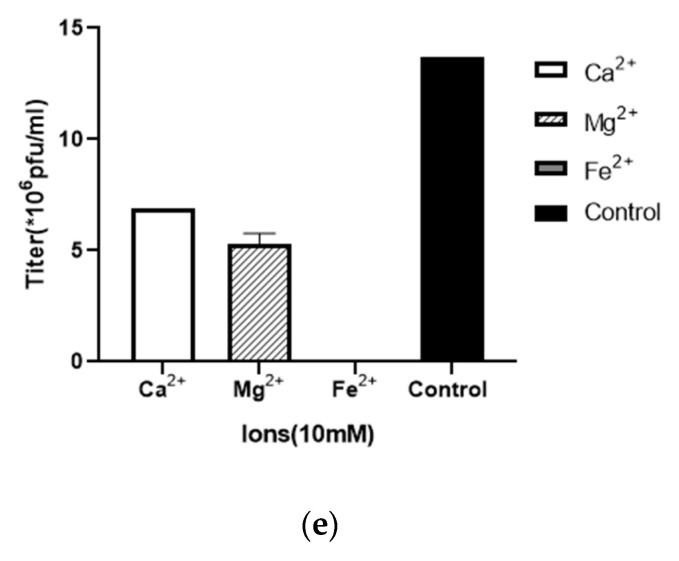
Cyanophage MinS1 stability tests under different conditions. (**a**) Thermal, (**b**) pH, and (**c**) saline concentrations; data points represent cyanophage titers and OD680 of mixture of phage and cyanobacteria. (**d**) UV and (**e**) ion sensitivity tests. All assays were performed in triplicate, and the control group data for pH, temperature, and NaCl content were the same as those of the experimental group with pH of 7, temperature of 25 °C, and NaCl content of 0%.

As a biocontrol agent with potential applications, the stability of the cyanophage was determined using both double-layer agar and liquid infection OD680 measuring methods under several stress conditions, including differing salinity, pH, UV, temperature, and metal ion levels. MinS1 tolerated a wide range of pH values from 3–12. However, extreme acidic conditions, such as pH 2, resulted in a loss of cyanophage activity (Figure 3a). After both 30 min and 60 min incubation times, the cyanophage MinS1 showed stability from 0 °C to 40 °C. In particular, at 0 °C, the phage was more stable with better activity, and this result also suggests that phages are more stable when stored at 0 °C. The activity of this phage was, however, lost following exposure to temperatures of 60 °C and higher (Figure 3b). After 1 h of incubation, MinS1 withstood a wide variation of salinities ranging from 1–20%, and the highest titer of phage was observed when NaCl concentrations were at 15% (Figure 3c). MinS1 was isolated from freshwater, yet it exhibited a preference for high salinity. We reviewed the salinity tolerance of some phages isolated from freshwater and found that not only MinS1 but also other phages, such as vB_ValP_IME234, have a high salinity tolerance [41]. However, further studies are needed to confirm why phages prefer salt. As shown in Figure 3d, UV irradiation had an effect on the activity of the phage, gradually decreasing activity levels as exposure time increased. In addition, MinS1 activity was affected by Fe^2+^, but it was tolerant to some extent when exposed to Ca^2+^ and Mg^2+^ (Figure 3e). The yellowing status of the phage infected with host cyanobacteria under several groups of different treatments is shown in Supplementary Figure S2. Our study is the first report on the stability of multiple environmental stresses on cyanophages. In conclusion, MinS1 showed varying degrees of tolerance to temperature, pH, UV, ions, and salinity, and these tolerance characteristics indicate that MinS1 might provide more opportunities for survival in an aquatic environment, suggesting that it could be an alternative biological approach toward inhibiting cyanobacterial growth and reducing the accumulation of microcystins. Besides this, the development of multi-cyanophage cocktails may have antagonistic effects on water blooms formed by multiple cyanobacterial outbreaks [42].

### 3.5. Genome Features

The cyanophage genome was sequenced (a 2 × 300 paired-end sequence method) on an Illumina MiSeq sequencing platform. MinS1 was found to be a GC-rich double-stranded DNA cyanophage, 49,966 bp long, with very low homology to known sequences within the NCBI database (1% homology coverage and 74.06% identity to the *Myoviridae* sp. ct6sY2). MinS1 had no obvious termini and no antibiotic tolerance genes, virulence genes or tRNAs.

**Figure 4:**
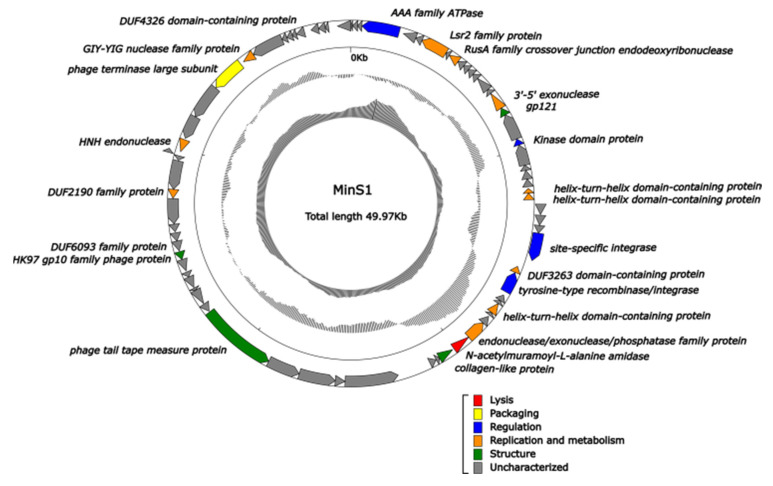
Genome map of the cyanophage MinS1 and functional annotation of its predicted proteins. The outermost circle represents 75 ORFs encoded within the genome, with different colors representing different functions (clockwise arrow indicates the forward reading frame, and counterclockwise arrow indicates the reverse reading frame); the gray circles in the middle represent the GC content (outwards indicates greater than the average GC content compared with the whole genome, and inwards indicates the opposite); the innermost circle represents the GC skew (G − C/G + C. Outwards indicates >0, and inwards indicates <0).

A total of 75 ORFs of MinS1 were predicted using RAST [18], averaging 203 amino acid (aa) long, the smallest of which had only 24 aa and the largest as many as 1155 aa (Table 3). Contrary to evidence from cyanophage SH-68 (107 positive strands and 10 negative strands), MinS1 had just 5 positive strands but 70 negative strands, but MinS1 had the same coding density (91.8%) as that of M. *elabens* vB_MelS-Me-ZS1. Homology alignments of the protein sequences (nr) database were applied to the 75 ORFs’ functional annotation; only 23 ORFs showed homology to genes with a known function, 39 were assigned to hypothetical proteins, and 13 had no homology to sequences within the database. This high rate of 69% of unknowns may be caused by the poor information known about the genomes of the cyanophages. Six functional modules (structure, replication, metabolism, regulation, packaging, and lysis) were divided among the MinS1 functionally annotated ORFs (Figure 4). The MinS1 genome therefore has similar functional modules to the other long-tailed phages and is capable of forming a complete phage independently.

**Lysis and packaging genes.** ORF38 and ORF65 are classified into lysis and packaging clusters, encoding N-acetylmuramoyl-L-alanine amidase and the terminase large subunit (TerL), respectively. In general, lysozyme homologs (e.g., lysin, holin, endolysin, and others) are commonly found in phages and are thought to be responsible for cell lysis [43,44,45]. However, the above lytic enzymes were not found, but N-acetylmuramoyl-L-alanine amidase was annotated within MinS1. In enzymology, N-acetylmuramoyl-L-alanine amidase (which belongs to the hydrolase family) is able to cleave the amide bond between N-acetylmuramoyl and L-amino acids in bacterial cell walls. Autolysins and some phage lysins are examples of N-acetylmuramoyl-L-alanine amidases [46]. Thus, ORF38, which encodes N-acetylmuramoyl and L-amino acid protein, may be the key gene responsible for lysis. This suggests that MinS1 adopts a different lysis strategy to others previously discovered, which may also be related to its broad-spectrum capabilities. TerL is commonly found in phages and is responsible for phage assembly. Due to its conserved nature, it is often used to aid evolutionary analysis.

**Structure and regulation genes.** The structure collection consists of ORF17, ORF39, ORF46, and ORF53, which encode gp121, collagen-like protein, tail tape measure protein (TMP), and gp10, respectively. Among these proteins, TMP determines the tail length and allows phage genes to transfer into the host [47]; however, this protein is not unique to tailed phages and has also been observed in tailless phages [48]. The regulation gene cassette consists of four ORFs, including AAA ATPase (ORF4), kinase domain protein (ORF19), site-specific integrase (ORF29), and tyrosine-type recombinase/integrase (ORF31). The AAA family proteins often perform chaperone-like functions, assisting in the assembly, operation, or disassembly of protein complexes [49].

**Replication and metabolism genes.** From an evolutionary perspective, phages have undergone multiple gene exchange events in response to selection pressure from their hosts, which has, in turn, driven their diversity [50]. Here, two ORFs associated with gene transfer were found in the MinS1 genome, HNH endonuclease (ORF62) and the GIY-YIG nuclease family protein (ORF66) [51,52,53]. They play a key role in relation to phages acquiring new genes when competing with specific bacteria, which facilitates survival adaptation [54]. The remaining ORFs in this block focus on nuclease activities, such as the endonuclease/exonuclease/phosphatase family protein (ORF37), which is involved in cellular signaling [55]; 3′-5′ exonuclease (ORF16), which controls the 3′-5′ exonuclease activity of DNA polymerase I and other enzymes and catalyzes mismatched nucleotide hydrolysis; the Lsr2 family protein (ORF7) and helix-turn-helix domain-containing protein (ORF24, ORF25, and ORF34), which are capable of multivariate regulation of gene expression and metabolism; and the RusA family crossover junction endodeoxyribonuclease (ORF9), which can correct DNA repair defects.

### 3.6. Phylogenetic Analysis

**Figure 5:**
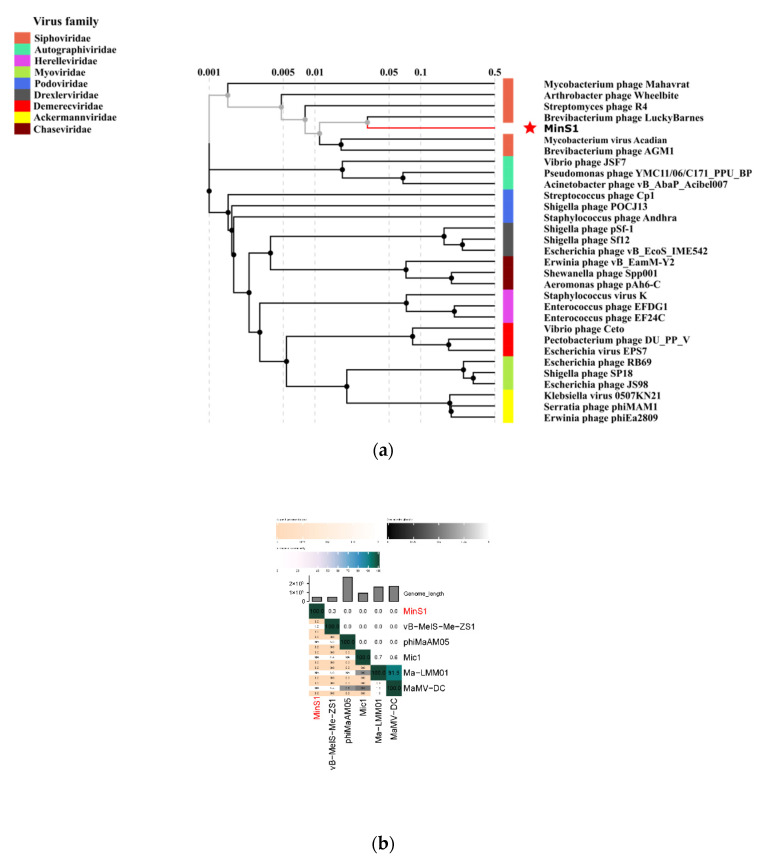
(**a**) Proteomic tree of the cyanophage MinS1 and 30 classified phages within Caudovirale. (**b**) Percent sequence similarity between the related Microcystis phages was calculated using VIRIDIC. The horizontal and vertical coordinates indicate the corresponding cyanophage, and the phage in this study is marked in red font.

To define the evolutionary status of newly isolated phages, sequences of TerL, capsid proteins, and other aspects are often used for phylogenetic analysis. However, the diversity of phage genomes increases with the number of sequenced sequences; hence, it is not accurate to simply classify them based on morphological features or single-gene evolutionary lineages alone. Whole-genome-based proteome phylogeny analysis is increasingly favored by researchers. The inter-genomic similarity calculated by VIRIDIC showed little similarity between MinS1 and all of the other known Microcystis cyanophages (Figure 5b). Thirty representative phage strains from the nine families belonging to Caudovirales in ICTV were selected, and a proteomic phylogenetic tree was constructed based on the MinS1 whole-genome sequence similarity. The proteomic tree (Figure 5a) showed that MinS1 was assigned into the cluster of *Siphoviridae* phages and was in the same branch with the Brevibacterium phage Lucky Barnes.

## 4. Conclusions

The present study described the characteristics and genome of MinS1, a novel strain of *Siphoviridae* freshwater cyanophage, with most of the predicted protein-coding genes showing no significant similarity to sequences within published databases. Its possible broad spectrum of genetic factors was explored through genomic analysis. In addition, MinS1 exhibited a broader host range than that of other known cyanophages and was tolerant to temperature, pH, UV, and salinity, suggesting that MinS1 has good potential for application as a biological control agent against cyanobacterial blooms. This research highlights our understanding of cyanophage biological characteristics, and it indicates that it has good potential for developing applications against water blooms caused by multiple cyanobacterial blooms.

## Figures and Tables

**Table 2:** Host range of MinS1 against 30 cyanobacteria strains.

Orders	Family	Species	Strains	Susceptibility	Toxin	Origin
*Chroococcales*	*Microcystaceae*	*Microcystis aeruginosa*	FACHB-905	+	*	China
			FACHB-942	−	*	China
			FACHB-469	+		France
			FACHB-924	−	*	Australia
			FACHB-1326	+		China
		*M. wesenbergii*	FACHB-908	+		China
			FACHB-1112	−		China
			FACHB-1317	+		China
			FACHB-1318	+		China
			FACHB-929	+	*	Japan
		*M. viridis*	FACHB-979	−	*	Japan
			FACHB-1342	+		China
			FACHB-1337	+		China
		*M. Flos-aquae*	FACHB-1028	−	*	China
			FACHB-1323	−		China
		*Microcystis* sp.	FACHB-915	+	*	France
		*M. elabens*	FACHB-916	−		Japan
	*Chroococcacaea*	*Chroococcus* sp.	FACHB-193	−		China
*Nostocales*	*Aphanizomenonaceae*	*Aphanizomenon flos-aquae*	FACHB-1040	−		China
		*Anabaena flos-aquae*	FACHB-245	+		America
		*Dolichospermum flos-aquae*	FACHB-1255	+		China
	*Nostocaceae*	*Nostoc* sp.	FACHB-596	+	*	China
*Oscillatoriales*	*Microcoleaceae*	*Planktothrix agardhii*	FACHB-1166	+		China
		*Planktothricoides raciborskii*	FACHB-881	+		China
	*Oscillatoriaceae*	*Oscillatoria planctonica*	FACHB-708	+		China
*Hormogonales*	*Scytonemataceae*	*Plectonema*	FACHB-402	+		America
			FACHB-240	−		America
*Synechococcales*	*Synechococcaceae*	*Synechococcus* sp.	FACHB-805	+		Australia
			FACHB-1061	+		China

Symbols: “+” suspective; “−” unsuspective; “*” means the strains can produce cyanotoxin according to references [39,40].

**Table 3:** ORF analysis of the MinS1cyanophage genome.

ORF	Size (aa)	Strand	Prediction Function	Top BLAST Hit ^a^	Identity ^b^ (aa)	Query Cover	E-Value ^c^
1	24	_	hypothetical protein	WP_193613138.1|Hypothetical protein [Nocardioides lijunqiniae]	18/27 (67%)	100%	4 × 10^−5^
2	75	_	hypothetical protein	YP_009950949.1|Hypothetical protein I5G75_gp45 [Mycobacterium phage Rando14]	26/59 (44%)	77%	6 × 10^−4^
3	64	_	No hit	No hit			
4	563	_	AAA family ATPase	WP_085894220.1|AAA family ATPase [*Nocardioides* sp. PD653]	384/552 (70%)	97%	0
5	196	_	hypothetical protein	WP_038679418.1|hypothetical protein [Pimelobacter simplex]	61/157 (39%)	77%	8 × 10^−24^
6	100	_	hypothetical protein	WP_085894217.1|hypothetical protein [*Nocardioides* sp. PD653]	59/100 (59%)	100%	7 × 10^−28^
7	403	_	Lsr2 family protein	WP_181312490.1|Lsr2 family protein [*Nocardioides* sp. Y192]	213/385 (55%)	94%	2 × 10^−135^
8	59	_	hypothetical protein	WP_091115340.1|hypothetical protein [Nocardioides psychrotolerans]	22/53 (42%)	89%	0.016
9	160	_	RusA family crossover junction endodeoxyribonuclease	WP_160006778.1|RusA family crossover junction endodeoxyribonuclease [*Nocardioides* sp. AX2bis]	106/157 (68%)	98%	9 × 10^−70^
10	107	_	hypothetical protein	WP_135361668.1|hypothetical protein [Mycolicibacterium peregrinum]	56/86 (65%)	80%	7 × 10^−29^
11	93	_	No hit	No hit			
12	97	_	No hit	No hit			
13	94	_	No hit	No hit			
14	225	_	hypothetical protein	WP_068120810.1|hypothetical protein [Nocardioides massiliensis]	144/225 (64%)	99%	1 × 10^−89^
15	72	_	No hit	No hit			
16	270	_	MULTISPECIES: 3′-5′ exonuclease	WP_165763480.1|MULTISPECIES: 3′-5′ exonuclease [unclassified Nocardioides]	140/271 (52%)	100%	6 × 10^−91^
17	118	_	gp121	NP_818419.1|gp121 [Mycobacterium virus Omega]	62/113 (55%)	95%	1 × 10^−33^
18	396	_	hypothetical protein	WP_160006801.1|hypothetical protein [*Nocardioides* sp. AX2bis]	260/403 (65%)	99%	2 × 10^−156^
19	95	_	Kinase domain protein	XP_001019386.2|kinase domain protein [Tetrahymena thermophila SB210]	26/84 (31%)	76%	0.003
20	313	_	hypothetical protein	WP_160006807.1|hypothetical protein [*Nocardioides* sp. AX2bis]	270/313 (86%)	100%	0.0
21	78	_	No hit	No hit			
22	132	_	hypothetical protein	NGZ99671.1|hypothetical protein [Nocardioides convexus]	53/131 (40%)	86%	7 × 10^−10^
23	115	_	No hit	No hit			
24	70	_	helix-turn-helix domain-containing protein	WP_091115310.1|helix-turn-helix domain-containing protein [Nocardioides psychrotolerans]	39/62 (63%)	88%	3 × 10^−22^
25	98	_	helix-turn-helix domain-containing protein	WP_191563416.1|helix-turn-helix domain-containing protein [Janibacter melonis]	52/91 (57%)	91%	6 × 10^−28^
26	144	+	hypothetical protein	WP_166844276.1|hypothetical protein [Pseudoteredinibacter isoporae]	65/131 (50%)	88%	2 × 10^−30^
27	139	+	hypothetical protein	WP_157346146.1|MULTISPECIES: hypothetical protein [unclassified Nocardioides]	76/132 (58%)	94%	2 × 10^−46^
28	107	+	hypothetical protein	WP_109746752.1|hypothetical protein [Salinispora mooreana]	44/73 (60%)	68%	3 × 10^−22^
29	393	+	site-specific integrase	GEP38839.1|site-specific integrase [Nocardioides psychrotolerans]	224/374 (60%)	94%	6 × 10^−130^
30	96	_	DUF3263 domain-containing protein	WP_020105292.1|DUF3263 domain-containing protein [*Nocardia* sp. 348MFTsu5.1]	42/73 (58%)	76%	2 × 10^−18^
31	314	_	tyrosine-type recombinase/integrase	NUO57292.1|tyrosine-type recombinase/integrase [*Hamadaea* sp.]	137/311 (44%)	98%	1 × 10^−74^
32	96	_	hypothetical protein	WP_182541196.1|hypothetical protein [Nocardioides ginsengisegetis]	48/93 (52%)	96%	3 × 10^−16^
33	51	_	hypothetical protein	WP_183407729.1|hypothetical protein [Marmoricola ginsengisoli]	26/50 (52%)	98%	5 × 10^−8^
34	167	_	helix-turn-helix domain-containing protein	WP_157537489.1|helix-turn-helix domain-containing protein [*Nocardioides* sp. Root190]	66/144 (46%)	81%	5 × 10^−25^
35	48	_	No hit	No hit			
36	130	_	hypothetical protein	WP_013861876.1|hypothetical protein [Microlunatus phosphovorus]	42/82 (51%)	63%	3 × 10^−17^
37	301	_	endonuclease/exonuclease/phosphatase family protein	MBA3989807.1|endonuclease/exonuclease/phosphatase family protein [Propionibacteriales bacterium]	69/249 (28%)	94%	1 × 10^−5^
38	278	_	N-acetylmuramoyl-L-alanine amidase	WP_067428568.1|N-acetylmuramoyl-L-alanine amidase [Nocardioides jensenii]	117/211 (55%)	75%	1 × 10^−64^
39	231	_	collagen-like protein	WP_091115432.1|collagen-like protein [Nocardioides psychrotolerans]	100/230 (43%)	95%	9 × 10^−27^
40	55	_	hypothetical protein	WP_170259192.1|hypothetical protein [Nocardioides psychrotolerans]	30/55 (55%)	100%	1 × 10^−12^
41	104	_	hypothetical protein	WP_143099800.1|hypothetical protein [Nocardioides psychrotolerans]	48/83 (58%)	79%	6 × 10^−25^
42	800	_	hypothetical protein	WP_179792624.1|hypothetical protein [Actinomycetospora corticicola]	133/421 (32%)	48%	7 × 10^−31^
43	148	_	hypothetical protein	WP_193613168.1|hypothetical protein [Nocardioides lijunqiniae]	37/95 (39%)	63%	1 × 10^−10^
44	548	_	hypothetical protein	WP_193613167.1|hypothetical protein [Nocardioides lijunqiniae]	276/548 (50%)	99%	4 × 10^−168^
45	491	_	hypothetical protein	WP_193613166.1|hypothetical protein [Nocardioides lijunqiniae]	175/492 (36%)	98%	3 × 10^−73^
46	1155	_	phage tail tape measure protein	WP_091115412.1|phage tail tape measure protein [Nocardioides psychrotolerans]	396/777 (51%)	64%	0.0
47	130	_	hypothetical protein	WP_091115411.1|hypothetical protein [Nocardioides psychrotolerans]	73/129 (57%)	96%	2 × 10^−38^
48	232	_	hypothetical protein	WP_091115408.1|hypothetical protein [Nocardioides psychrotolerans]	103/228 (45%)	97%	1 × 10^−57^
49	39	_	No hit	No hit			
50	173	_	hypothetical protein	WP_085894250.1|hypothetical protein [*Nocardioides* sp. PD653]	109/169 (64%)	97%	2 × 10^−72^
51	75	_	hypothetical protein	WP_085894249.1|hypothetical protein [*Nocardioides* sp. PD653]	52/76 (68%)	100%	6 × 10^−26^
52	179	_	hypothetical protein	WP_085894248.1|ypothetical protein [*Nocardioides* sp. PD653]	87/178 (49%)	79%	2 × 10^−40^
53	121	_	HK97 gp10 family phage protein	WP_068125082.1|HK97 gp10 family phage protein [Nocardioides massiliensis]	80/121 (66%)	100%	5 × 10^−50^
54	151	_	DUF6093 family protein	WP_091115395.1|DUF6093 family protein [Nocardioides psychrotolerans]	77/153 (50%)	98%	5 × 10^−29^
55	124	_	hypothetical protein	WP_091115392.1|hypothetical protein [Nocardioides psychrotolerans]	82/121 (68%)	95%	6 × 10^−47^
56	114	_	hypothetical protein	WP_193613156.1|hypothetical protein [Nocardioides lijunqiniae]	66/113 (58%)	98%	5 × 10^−25^
57	376	_	hypothetical protein	WP_193613155.1|hypothetical protein [Nocardioides lijunqiniae]	311/374 (83%)	99%	0.0.
58	133	_	DUF2190 family protein	WP_193613154.1|DUF2190 family protein [Nocardioides lijunqiniae]	111/136 (82%)	100%	2 × 10^−64^
59	430	_	hypothetical protein	WP_220138645.1|hypothetical protein [Nocardioides massiliensis]	210/352 (60%)	81%	4 × 10^−127^
60	63	_	No hit	No hit			
61	57	+	hypothetical protein	NUR90848.1|hypothetical protein [*Nonomuraea* sp.]	40/55 (73%)	94%	5 × 10^−15^
62	186	_	HNH endonuclease	WP_114027590.1|HNH endonuclease [Sphaerisporangium album]	89/179 (50%)	97%	6 × 10^−43^
63	353	_	hypothetical protein	WP_091115383.1|hypothetical protein [Nocardioides psychrotolerans]	224/350 (64%)	99%	1 × 10^−154^
64	525	_	hypothetical protein	WP_193613151.1|hypothetical protein [Nocardioides lijunqiniae]	393/528 (74%)	99%	0.0
65	508	_	phage terminase large subunit	WP_210651751.1|phage terminase large subunit [*Nocardioides* sp. SYSU D00065]	382/516 (74%)	98%	0.0
66	179	_	GIY-YIG nuclease family protein	WP_131823145.1|GIY-YIG nuclease family protein [*Mycolicibacterium* sp. (ex *Dasyatis americana*)]	58/167 (35%)	93%	7 × 10^−18^
67	485	_	hypothetical protein	WP_193613147.1|hypothetical protein [Nocardioides lijunqiniae]	265/473 (56%)	97%	1 × 10^−166^
68	77	_	hypothetical protein	WP_183591126.1|hypothetical protein [Nocardioides soli]	37/84 (44%)	100%	1 × 10^−6^
69	88	_	No hit	No hit			
70	63	_	No hit	No hit			
71	125	_	DUF4326 domain-containing protein	WP_191008157.1|DUF4326 domain-containing protein [Microbacterium hominis]	55/117 (47%)	93%	3 × 10^−26^
72	115	_	hypothetical protein	WP_091115362.1|hypothetical protein [Nocardioides psychrotolerans]	63/86 (73%)	74%	6 × 10^−36^
73	99	_	hypothetical protein	WP_085894225.1|hypothetical protein [*Nocardioides* sp. PD653]	84/93 (90%)	93%	4 × 10^−55^
74	51	_	No hit	No hit			
75	199	_	hypothetical protein	WP_068121038.1|hypothetical protein [Nocardioides massiliensis]	139/202 (69%)	100%	4 × 10^−87^

Symbol: ^a^ the most closely related protein and its organism. ‘‘No hits’’ indicates no significant hits detected for a particular amino acid sequence. ^b^ percentage identity for the top hits in BLASTP searches. Numbers in parentheses represent the length of each alignment. ^c^ probability of obtaining a match by chance as determined by BLASTP analysis.

## Data Availability

Not applicable.

## Supplementary Materials

The following supporting information can be downloaded at: https://www.mdpi.com/article/10.3390/v14020433/s1, Figure S1: (a) Phenotypic photographs of cyanobacterial cultures within the host range experiments. (b) Phage spots formed by the cyanophage MinS1 on susceptible cyanobacteria strains; Figure S2: Status of host cyanobacteria infected by MinS1 treated under different conditions. Table S1: MinS1 classification, general features, and genome sequencing information.
