# Supplementary material for: A Novel Wide-Range Freshwater Cyanophage MinS1 Infecting the Harmful Cyanobacterium Microcystis aeruginosa
Source: Viruses. 2022 Feb 20;14(2):433. doi: 10.3390/v14020433 (PMC8876498; doi:10.3390/v14020433)
Supplement: Supplementary file 1 [file viruses-14-00433-s001.zip › viruses-1571525-supplementary.pdf]

Supplementary Figures

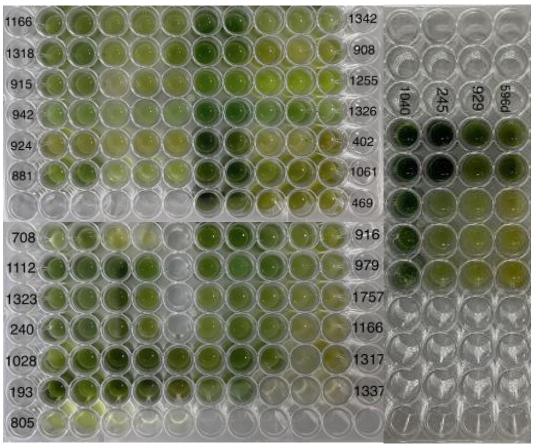

(**a**)

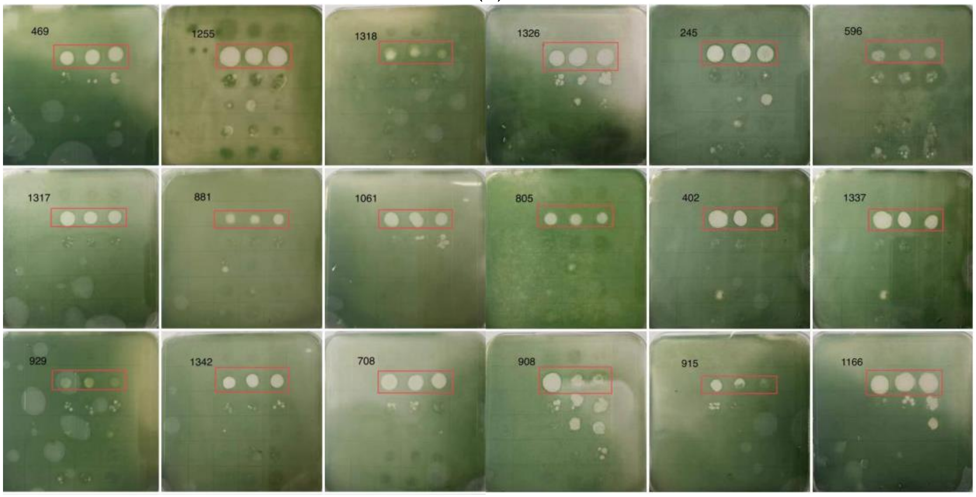

(**b**)

Figure S1: (a) Phenotypic photographs of cyanobacterial cultures within the host range experiments. (b) Phage spots formed by the cyanophage MinS1 on susceptible cyanobacteria strains.

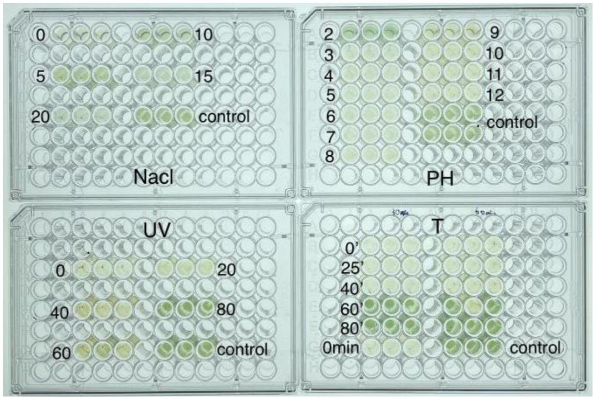

Figure S2: Status of host algae infected by MinS1 treated under different conditions.

Supplementary Tables

Table S1: MinS1 classification, general features, and genome sequencing information.

Item	Description
Classification	Order Caudovirales, Family Siphoviridae
Particle shape	isometric hexagon head with a long non-contractile tail
Submitted to GenBank	MZ923504
Investigation type	Virus
Geographic location	Mayang Stream, Fujian, China
Altitude, latitude and longitude	13m, 24°32′37.81″ N, 117°46′26.40″ E
Depth	0.2m
Collection date and time	November 23, 2020 at 10:17 am
Temperature	20°C
Environment (material)	Freshwater
Sequencing method	Illumina Miseq
Number of contigs	1
Assembly method	SPAdes 3.13.0
Finishing quality	Finish (complete)
